# Supplementary material for: Structural basis of ClC-3 transporter inhibition by TMEM9 and PtdIns(3,5)P2
Source: Nat Struct Mol Biol. 2025 Jul 16;32(10):1972–9. doi: 10.1038/s41594-025-01617-2 (PMC12403049; doi:10.1038/s41594-025-01617-2)
Supplement: Supplementary file 1 — Reporting Summary [file 41594_2025_1617_MOESM1_ESM.pdf]

nature portfolio

Corresponding author(s): Hite, Richard K
Jentsch, Thomas J

Last updated by author(s): May 27, 2025

Reporting Summary

Nature Portfolio wishes to improve the reproducibility of the work that we publish. This form provides structure for consistency and transparency in reporting. For further information on Nature Portfolio policies, see our Editorial Policies and the Editorial Policy Checklist.

Statistics

For all statistical analyses, confirm that the following items are present in the figure legend, table legend, main text, or Methods section.

n/a	Confirmed	
☐	☒	The exact sample size (*n*) for each experimental group/condition, given as a discrete number and unit of measurement
☐	☒	A statement on whether measurements were taken from distinct samples or whether the same sample was measured repeatedly
☐	☒	The statistical test(s) used AND whether they are one- or two-sided<br>*Only common tests should be described solely by name; describe more complex techniques in the Methods section.*
☒	☐	A description of all covariates tested
☒	☐	A description of any assumptions or corrections, such as tests of normality and adjustment for multiple comparisons
☐	☒	A full description of the statistical parameters including central tendency (e.g. means) or other basic estimates (e.g. regression coefficient) AND variation (e.g. standard deviation) or associated estimates of uncertainty (e.g. confidence intervals)
☐	☒	For null hypothesis testing, the test statistic (e.g. *F*, *t*, *r*) with confidence intervals, effect sizes, degrees of freedom and *P* value noted<br>*Give P values as exact values whenever suitable.*
☒	☐	For Bayesian analysis, information on the choice of priors and Markov chain Monte Carlo settings
☒	☐	For hierarchical and complex designs, identification of the appropriate level for tests and full reporting of outcomes
☒	☐	Estimates of effect sizes (e.g. Cohen's *d*, Pearson's *r*), indicating how they were calculated

Our web collection on statistics for biologists contains articles on many of the points above.

Software and code

Policy information about availability of computer code

Data collection	SerialEM 4.2, EPU 3.9.1, CryoSparc Live v4.2.1
Data analysis	CryoSparc v3.2.0, CryoSparc v4.2.1, PyEM (https://doi.org/10.5281/zenodo.3576630), Clustal Omega (1.2.3) Relion v3.1.3, Phenix v1.21.1-5286, Coot 0.9.6, PyMol (Schrodinger, LLC. 2010. The PyMOL Molecular Graphics System, Version 2.5.3), ChimeraX 1.5, GraphPad Prism 9, MOLE, ImageJ

For manuscripts utilizing custom algorithms or software that are central to the research but not yet described in published literature, software must be made available to editors and reviewers. We strongly encourage code deposition in a community repository (e.g. GitHub). See the Nature Portfolio guidelines for submitting code & software for further information.

Data

Policy information about availability of data

All manuscripts must include a data availability statement. This statement should provide the following information, where applicable:

- Accession codes, unique identifiers, or web links for publicly available datasets
- A description of any restrictions on data availability
- For clinical datasets or third party data, please ensure that the statement adheres to our policy

Cryo-EM maps have been deposited in the EMDB under accession codes EMD-47070 [https://www.ebi.ac.uk/emdb/EMD-47070] (ClC-3), EMD-47066 [https://www.ebi.ac.uk/emdb/EMD-47066] (ClC-3/noT9A), EMD-47067 [https://www.ebi.ac.uk/emdb/EMD-46067] (ClC-3/T9A, T9A Protomer A and B: Complete),

EMD-47068 [https://www.ebi.ac.uk/emdb/EMD-47068] (ClC-3/T9A, T9A Protomer A: No CD, T9A Protomer B: No LD, No CD) and EMD-47069 [https://www.ebi.ac.uk/emdb/EMD-47069] (ClC-3/T9A, T9A Protomer A: Complete, T9A Protomer B: No LD, No CD). Atomic coordinates have been deposited in the PDB under accession codes 9DO0 [https://doi.org/10.2210/pdb9DO0/pdb] (ClC-3), 9DNW [https://doi.org/10.2210/pdb9DNW/pdb] (ClC-3/no T9A), 9DNX [https://doi.org/10.2210/pdb9DNX/pdb] (ClC-3/T9A, T9A Protomer A and B: Complete), 9DNY [https://doi.org/10.2210/pdb9DNY/pdb] (ClC-3/T9A, T9A Protomer A: No CD, T9A Protomer B: No LD, No CD) and 9DNZ [https://doi.org/10.2210/pdb9DNZ/pdb] (ClC-3/T9A, T9A Protomer A: Complete, T9A Protomer B: No LD, No CD). The atomic coordinates of previously published structures of bovine ClC-K [https://doi.org/10.2210/pdb5TQQ/pdb], human ClC-2 [https://doi.org/10.2210/pdb8TA4/pdb], human ClC-6 [https://doi.org/10.2210/pdb8JPJ/pdb], and human ClC-7/OSTM1 complex [https://doi.org/10.2210/7JM7/pdb] were used in this study.

Human research participants

Policy information about studies involving human research participants and Sex and Gender in Research.

Reporting on sex and gender	N/A
Population characteristics	N/A
Recruitment	N/A
Ethics oversight	N/A

Note that full information on the approval of the study protocol must also be provided in the manuscript.

Field-specific reporting

Please select the one below that is the best fit for your research. If you are not sure, read the appropriate sections before making your selection.

☒ Life sciences ☐ Behavioural & social sciences ☐ Ecological, evolutionary & environmental sciences

For a reference copy of the document with all sections, see nature.com/documents/nr-reporting-summary-flat.pdf

Life sciences study design

All studies must disclose on these points even when the disclosure is negative.

Sample size	Sample sizes were not predetermined with statistical means but based on standard numbers in the field. Cryo-EM sample size was determined by the availible microscope time. The number of images collected is indicated in Extended Data Figure 2.
Data exclusions	Cryo-EM images were excluded from the data set when they showed evidence of high drift or poor CTF fits. Individual particles were excluded by 2D and 3D classification as is the standard in the field of single-particle cryo-EM analysis. The selection of particles is shown in Extended Data Figure 2.<br>For the CLC vacuolization assay, only cells which displayed above-background fluorescence in both the Venus- and the T9 channel (about 20-25% of total cells) were manually selected for further analysis.
Replication	Cryo-EM: Each condition was imaged from one or two grids, with the results being similar.<br>All CLC vacuolization experiments were independently performed two times, with the results being similar.
Randomization	Cryo-EM particles were randomized during image processing to calculate FSC curves.<br>No randomization was done for vacuolization assays.
Blinding	Blinding was not performed as cryo-EM image analysis requires careful evaluation at step of the image processing workflow to ensure that high-quality reconstructions are obtained.<br>No blinding was performed for vacuolization assays, but fields randomly chosen. Several fields were imaged per preparation and by different researchers to avoid acquisition bias.

Reporting for specific materials, systems and methods

We require information from authors about some types of materials, experimental systems and methods used in many studies. Here, indicate whether each material, system or method listed is relevant to your study. If you are not sure if a list item applies to your research, read the appropriate section before selecting a response.

Materials & experimental systems

n/a	Involved in the study	
☐	☒	Antibodies
☐	☒	Eukaryotic cell lines
☒	☐	Palaeontology and archaeology
☒	☐	Animals and other organisms
☒	☐	Clinical data
☒	☐	Dual use research of concern

Methods

n/a	Involved in the study	
☒	☐	ChIP-seq
☒	☐	Flow cytometry
☒	☐	MRI-based neuroimaging

Antibodies

Antibodies used

chicken anti-GFP antibody (1: 500; Cat# GFP-1020, Aves Lab)
guinea pig anti-T9A (T9AC2) (1:1000; Pineda Antibody Service, Berlin)
mouse anti-Lamp-2 (H4B4) (1:500; Cat. Ab25631, Abcam)
Goat anti-chicken Alexa 488 Cat. A11039
Goat anti-guinea pig Alexa 555 Cat. A21435
goat anti-mouse Alexa 633 Cat. A21052, Invitrogen

Validation

Validation for anti-T9A antibody is presented in reference 29.

Eukaryotic cell lines

Policy information about cell lines and Sex and Gender in Research

Cell line source(s)

HEK293S GnTI- (ATCC CRL-3022)
Expi293F (Gibco)
HeLa (Leibniz-Institut DSMZ- Deutsche Sammlung von Mikroorganismen und Zellkulturen GmbH, Germany)
HeLa ClC-7 KO (reference 58)

Authentication

Cells were authenticated by the Deutsche Sammlung von Mikroorganismen und Zellkulturen, Germany

Mycoplasma contamination

Cells were regularly tested for contamination by PCR

Commonly misidentified lines
(See ICLAC register)

No commonly misidentified cell lines were used in this study.
