# Supplementary figures and images for: Structural basis of ClC-3 transporter inhibition by TMEM9 and PtdIns(3,5)P2
Source: Nat Struct Mol Biol. 2025 Jul 16;32(10):1972–9. doi: 10.1038/s41594-025-01617-2 (PMC12403049; doi:10.1038/s41594-025-01617-2)

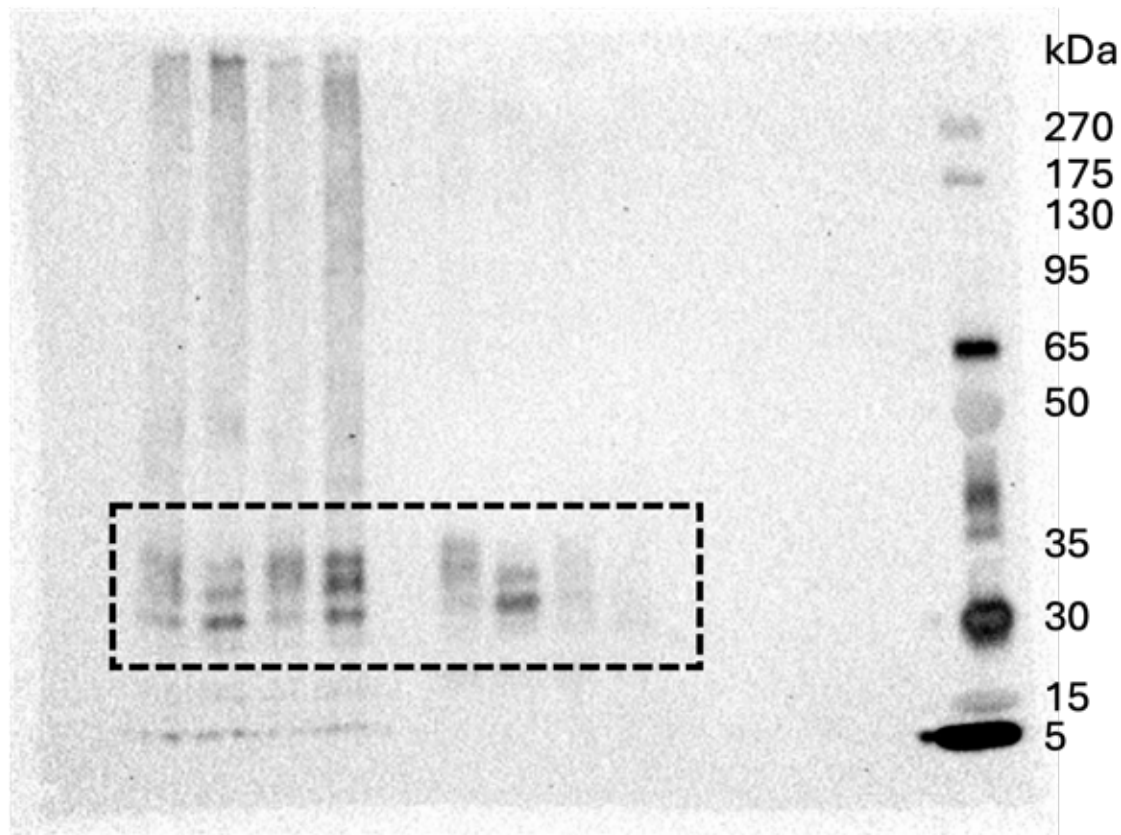

Supplement: Supplementary file 6 — Uncropped blot. [file 41594_2025_1617_MOESM6_ESM.pdf]
